# A Comparative Study of Corneal Topography in Children with Autism Spectrum Disorder: A Cross-Sectional Study

**DOI:** 10.3390/vision5010004

**Published:** 2021-01-15

**Authors:** Maha A. ALGarzaie, Ali M. Alsaqr

**Affiliations:** 1 Department of Optometry, Prince Sultan Military Medical City, Riyadh 091510, Saudi Arabia; opto_911@windowslive.com; 2 Department of Optometry, College of Applied Medical Sciences, King Saud University, Riyadh 091510, Saudi Arabia

**Keywords:** autism, corneal power, corneal descriptors, keratoconus, corneal shape

## Abstract

Purpose: To investigate the corneal characteristics in individuals with autism spectrum disorder (ASD) and age-matched typical development (TD) participants. Methods: This cross-sectional, clinically based study compared children with ASD to age-matched TD participants. Corneal topography was measured with a portable EyeSys Vista system. The distance visual acuity (VA) and the contrast sensitivity (CS) were determined. The refractive error (RE) was assessed using a 2WIN autorefractometer. Results: A total of 31 children with ASD (mean age: 12.78 ± 4.49 years), and 60 participants with TD (mean age: 13.65 ± 3.56 years) were recruited. The two groups were similar in age (t = −2.084, *p* = 0.075) and VA (t = −0.35, *p* = 0.32). Most of the children with ASD had a significant amount of refractive errors (REs; range: +5.25 to −5.50 DS), and astigmatism was dominant (range: −0.25 to −4.50 DC). There was no statistically significant difference between both groups in terms of average corneal power (t = 1.12, *p* = 0.39). The children with ASD and participants with TD also did not differ significantly in terms of corneal shape descriptors (*p* > 0.05), such as corneal asphericity, inferior superior index, opposite sector index, and differential sector index. The spherical equivalent did not differ significantly between the ASD participants and participants with TD (t = 1.15, *p* = 0.15). There was a significant difference (*p* < 0.05) in the astigmatism component between the ASD participants and the participants with TD.

## 1. Purpose

Autism spectrum disorder (ASD) comprises a group of developmental disabilities characterized by impairments in social interaction and communication, and by restricted, repetitive, and stereotyped patterns of behavior [1]. It was reported that most children with ASD have sensorimotor abnormalities that may lead to social communication and cognitive deficits early in life [2]. The symptoms are observed between 2–4 years of age [3]. However, in other cases, the symptoms of ASD may start as early as 18 months of age, involving the inability to look when the child’s name is called and a minimal interest of the patient in interacting with other children [4]. ASD occurs four times more frequently in males than in females [5]. The frequency of ASD in Saudi Arabia has been reported to be 1.8 per 1000 children [6].

The reported ophthalmic-related behavioral characteristics of children with ASD include poor or no eye contact, gaze abnormalities, and impairment of facial recognition, which has been related to disturbances of eye movements [7,8]. Many studies have reported from observations of children with autism that individuals with ASD have abnormal oculomotor functions [9,10]. Most of them have hypometric saccadic movements and difficulties in performing smooth pursuit eye movements and low velocity movements. These findings were linked to brainstem dysfunction in autism [9]. 

The visual acuity in children with ASD was investigated in a number of studies [11,12,13,14,15,16]. The majority that have been previously reviewed reported that children with ASD apparently have normal visual acuity (VA) [17]. Contrast sensitivity (CS), or the ability to distinguish differences between an object and its background, was studied in children with ASD and compared in several studies to a matched control group. Some studies reported that there were no difference between ASD and normal participants [18,19], while other studies suggested that participants with ASD have reduced CS thresholds [20,21]. The diversity of these reports can be partly due to the wide variation in methods used to measure CS [17]. However, these studies indicated early visual sensory processing deficits associated with autism [17]. The color perception among participants with ASD has been reported to be poor. There is a general reduction in color sensitivity detection, rather than specific deficiencies of color perception [22,23].

A previous study reported that most children of ages 1–14 years with autism had hyperopia (farsightedness), oblique astigmatism (the eye does not focus light evenly on the back of the eye), and exotropia (type of strabismus) [24]. It was also reported that the amount and prevalence of refractive astigmatism was significantly higher among European Caucasian children with ASD (mean age: 10.9 ± 3.3 years) than among typically developed children with a mean age of 11.5 ± 3.1 years [25]. Most previous studies that investigated visual abnormalities among children with ASD were focused on oculomotor functions, color perception, binocular vision, and refractive errors (REs) [19,22,23,24,25]. A regular checkup at an early age is very important to provide the best treatment to prevent the progression and complications of keratoconus. Keratoconus is a non-inflammatory, mostly asymmetric, progressive disease, resulting in corneal thinning, irregular astigmatism, and eventually severe vision loss [26]. Worldwide, the prevalence of keratoconus ranges from 0.17 to 40 in 1000; this very wide range could be accounted to differences in genetic factors, the locations of study, environmental exposure, the methodology and design of the studies, and diagnostic criteria and instruments [26]. To the best of our knowledge, there is no published study that has examined corneal parameters as assessed using topography for children with ASD in order to determine if they have higher/lower risk of keratoconus.

This study was conducted to compare several corneal shape descriptors, including corneal power, steepest and flattest K-reading, corneal asphericity (Q-value), inferior superior index (IS), opposite sector index (OSI), differential sector index (DSI), and center/surround index (CSI) between children with ASD and typical development (TD) participants. These descriptors are the main determinates of the corneal power, shape, and integrity [27,28,29]. The change in these descriptors from what would be found in the TD group may indicate that the ASD group is at higher risk of developing keratoconus. This study also compared VA, CS, and REs between ASD and participants with TD. The findings of this study can provide insights for optometrists and other healthcare professionals, parents, advocates, and school administrators in providing special care for this subgroup of individuals.

## 2. Material and Methods 

This was a prospective cross-sectional-based clinical study. The recruited participants had documented diagnoses of ASD and were compared with TD participants. Any participant with corneal opacities, corneal scars, cataracts, or any previous ocular surgery was excluded from the study.

The study recruited children with ASD attending the general clinic at 5 autism specialist centers. A psychiatrist who specialized in ASD working in those centers diagnosed the recruited children with ASD. Diagnostic tools including autism diagnostic observation schedule (ADOS) and autism diagnostic interview-revised (ADI-R) are widely used in Saudi Arabia for confirming the ASD, however, the author collecting the data did not include this information but made sure that the recruited participants were confirmed children with ASD. Forty-five invitations were sent to the parents of children with ASD who attended the general clinic at the 5 specialist centers. The response rate was 70%. Three participants with ASD were excluded from the study due to their poor cooperation for optometric measurements. The 60 participants with TD were recruited from the ophthalmology clinic at Prince Sultan Military Medical City, Riyadh, Saudi Arabia. In terms of the racial background of the participants with ASD and TD, the participants were Saudis with Arab background.

The distance VA was measured using a Lea Numbers chart (Good-Lite, Elgin, IL, USA), which was placed 3 m in front of the participant in a well-lit room. The monocular VA was measured by occluding the patient’s eye with an occluder and determining the LogMAR. Each letter had a score of 0.02 log units. The VA score was calculated as follows: LogMAR VA = 0.1 + LogMAR value of the best line read—0.02 (number of optotypes read) [30].

The CS was measured using a Mars Numeral CS chart (Mars Perceptrix, Chappaqua, NY, USA). The chart was held at approximately 50 cm with habitual correction, when necessary, in a well-lit room (the illumination level was ≈85 cd/m^2^). The participant was asked to read the numerals across the lines and down the chart. The CS of the final numerals before the patient misidentified 2 consecutive numerals, with a correction for earlier incorrect responses, determined the logCS. The test was conducted both monocularly and binocularly. The Mars test produces results with excellent agreement and reliability and is considered equivalent to the Pelli–Robson test [31].

The REs were assessed using a 2WIN binocular handheld refractometer and a vision analyzer (Adaptica, Padova, Italy). The apparatus measured both eyes at the same time in a natural vision situation. The 2WIN refractometer can detect REs and visual misalignment. It has been reported that the REs of children 1–18 years of age measured using a 2WIN refractometer without cycloplegia were similar in spherical power and spherical equivalent (SE) values to those obtained using cycloplegic retinoscopy [32]. The astigmatism was calculated using power vector notation (J_0_ and J_45_) and by applying a Fourier transformation [33].

Keratoconus risk factor assessment was conducted using the corneal topography (elevation-based topography) and asphericity coefficient with an EyeSys Vista system (Eye Sys Vision, Houston, TX, USA). The EyeSys Vista is a placido-based videokeratography device with 25 rings and measurement was taken in a 3 mm zone. It has been suggested that this device can be used in most clinical settings [34], and its corneal curvature measurements exhibit good reproducibility [35]. The key corneal measurements included the following: simulated K-readings (steepest K and flattest K) representing corneal astigmatism; the asphericity (Q-value), which describes the rate of curvature variation of the cornea from its center to the periphery and specifies the type of conicoid that best represents its shape [27]; the inferior superior index (I-S), which refers to inferior-superior dioptric asymmetry as obtained by averaging the superior corneal power (meridians of 30°, 60°, 90°, 120°, 150°) and subtracting that value from the average inferior corneal power (meridians of 210°, 240°, 270°, 300°, and 330°) [14], with a difference of > 1.4 diopters (D) being indicative of keratoconus [28]; OSI, opposite sector index; DSI, differential sector index; CSI, center/surround index (OSI > 2 D, DSI > 2.4, and CSI > 1 indicative of keratoconus) [29]. The OSI and DSI were a result of dividing the corneal area into eight pie-shaped sectors; the mean corneal power was also calculated for each sector. The DSI is the greatest difference of the mean corneal power between any 2 sectors. The OSI is the maximum difference between mean corneal powers in opposite sectors. The CSI is the difference between the mean corneal power of the central area and the annulus surrounding the central area. More details of the calculation method of OSI, DSI, and CSI can be found in the study by Maeda et al. [29]. The horizontal visible iris diameter (HVID) refers to the horizontal diameter of the iris within the clear corneal zone [36], and the vertical palpebral aperture (PA) is the distance between the inferior margin of the eyelid to the superior eyelid margin over the pupil [37]. These parameters are the main corneal parameters that have been previously suggested to be the earliest indicators of corneal changes in early/subclinical keratoconus [27,28,29]. One eye from each participant, in both groups, was randomly selected in order to record the corneal and visual measurements. This was to avoid any bias may be caused as a result of any eventual correlation existing between right and left eyes of a single patient [38,39].

The IBM SPSS Statistics for Windows, version 22 (IBM Corp., Armonk, NY, USA), was used in the data analysis. The data were normally distributed (Kolmogorov–Smirnov test, *p* > 0.05), and therefore the mean ± standard deviation was used to report the data. Further, the independent *t*-test was also used to investigate the differences between the two groups. The differences were considered statistically significant when the *p*-value was < 0.05.

## 3. Results

A total of 30 of 31 children with ASD completed the refraction and the corneal topography assessments (one was excluded due to poor image quality). Those children did not have any previous ocular or medical history/diagnosis. The VA and best-corrected VA were measured in 26 of 31 children with ASD, and the CS was determined in 20 of 31 children with ASD. The inability to perform these visual functions was due to the inability of the children to concentrate. All measurements were compared with those of participants with TD. The mean ages of the children with ASD (mean age: 12.78 ± 4.49 (standard deviation) years; range: 5–20 years) and the participants with TD (mean age: 13.65 ± 3.56 years; range: 6–20 years) did not differ significantly (Table 1).

The mean unaided VA in the children with ASD and participants with TD was 0.28 ± 0.28 and 0.33 ± 0.37 LogMAR, respectively. The unaided VA did not differ significantly between the children with ASD and the participants with TD (*p* > 0.05) (Table 1). The corrected VA in children with ASD was similar to that in participants with TD with means of 0.03 ± 0.08 and 0.03 ± 0.03 LogMAR, respectively. 

Strabismus was observed only in two children with ASD. One patient presented with esotropia with spectacle correction, whereas the other presented with exotropia without spectacle correction. None of the participants with TD had any ocular deviation.

The descriptive/frequency distribution of REs was different between the children with ASD and participants with TD, whereas the CS of the ASD and participants with TD were similar (Table 2). No significant differences were found in the visual functions (Table 1). However, the astigmatism vector analysis showed that there was a significant difference between both groups (Table 1), with the ASD group having higher astigmatism scores.

### Corneal Parameters

The mean steep- and flat-simulated K-readings of the children with ASD were slightly steeper than those of the participants with TD. However, these small differences were not statistically significant (Table 3). There was no significant difference in the mean delta-K between children with ASD and participants with TD. The mean average simulated K of the children with ASD was similar to that of the participants with TD (Table 3).

The comparison of the corneal parameters presented in Table 3 showed that, in general, there were no significant differences between both groups. However, the ASD group had a narrower vertical PA compared to the participants with TD. There was a statistically significant differences in the PA size between the ASD group and the participants with TD (Table 3). The mean HVID in the ASD group was slightly less than that in the TD group, and the differences were statistically significant (Table 3). In detail, there were no significant differences in the Q-values between children with ASD and participants with TD (Table 3). The mean Q-values of the children with ASD were similar to those of the participants with TD. Both groups had negative Q-values, which indicated a prolate cornea (Table 3). The mean inferior–superior (IS) dioptric power of both eyes in both groups was <1.4 D, which was within normal limits (Table 3). In detail, the number of cases in the ASD and TD group that had been observed to have IS scores >1.4 were 7 and 12 cases, respectively. Those cases were not classified as keratoconus cases as the K reading was less than 45.25 D. The OSI, DSI, and CSI in both groups are shown in Table 3. These indices did not differ significantly between groups and were less than the cut off points for suspected keratoconus suggested previously [29].

## 4. Discussion

This study was one of the first to compare the corneal parameters of children with ASD with those of participants with TD, providing indicative preliminary results. There have been plenty of studies that have reported the prevalence of keratoconus in different populations [26,40]. The prevalence in Saudi Arabia was reported to be 1:26 [41], although larger scale population studies are needed to confirm this large prevalence outcome in comparison to other geographical locations. Unfortunately, to date, no published report has been found in terms of the prevalence of keratoconus in children with ASD. Therefore, the range of possible keratoconus rate differences between typical healthy participants and children with ASD is out of reach of the discussion at this stage. 

Although the response rate of children with ASD was high, approximately 30% of the children did not think that they could participate in this study. The children with ASD had smaller HVIDs and PA sizes compared with the participants with TD, and these differences were significantly different. The differences could have been due to differences in facial characteristics between children with ASD and participants with TD, with the former exhibiting an abnormal slant of palpebral fissures, wide nasal bridges, thin vermilion of the upper lips, small low-set ears, and protruding cup-shaped ears [42]. Palpebral fissure characteristics are clinically important in areas such as contact lens fitting [43].

There were no significant differences in the corneal power (steep and flat K-readings) between the two groups. The mean average K-readings were slightly steeper in the children with ASD compared with the participants with TD, but this was not statistically significant. The smaller PA of children with ASD may have affected corneal curvature as well. These results could preliminarily indicate that there were no significant clinical differences between the two groups.

In addition, the asphericity (Q-value), OSI, DSI, and CSI did not differ significantly between the two groups, indicating that it is unlikely that there was a difference between the two groups. However, there were some participants in the ASD and TD groups whose IS scores were >1.4 (7 ASD participants and 12 participants with TD). No subclinical keratoconus was confirmed nor reported in the result, as the steep K reading in those participants was <45.25 D. Further, the steep/flat K readings in children with ASD were within normal limits (the steepest K reading scores were <47 D). Furthermore, the mean IS dioptric power in both groups was <1.4 D, which is not indicative of keratoconus [28]. However, the reason for the higher IS scores in those participants was not conclusive, and one of the explanations might be eye rubbing. A previous report suggested that children with keratoconus are more likely to be eye rubbers, and to have associated allergic diseases [44]. In future study, the history and habits of eye rubbing and allergic diseases should be included in demographic data collection. One study reported a relationship between atopic diseases and ASD, although this finding remains debatable [45]. The REs were also measured in children with ASD and participants with TD. Most children with ASD had compound hyperopic astigmatism followed by compound myopic astigmatism, whereas most participants with TD had compound myopic astigmatism. The cylindrical components (J_0_ and J_45_) of children with ASD were significantly higher than those of participants with TD (Table 2). Furthermore, the mean SE did not differ significantly between the two groups. A recent study by Anketell and his colleagues also found that there were no significant differences in SE between a large population of European Caucasian children with ASD (mean age: 10.9 ± 3.3 years; range: 6.4–16.50 years) and typically developed children (mean age: 1.5 ± 3.1 years; range: 5–18 years) [25]. The REs in their study were assessed using a cycloplegic agent with an autorefractor. In the present study, the mean SE for participants with TD (OD or right eye: –0.76 ± 1.66) was less than that reported by Lim et al. for a large population of Singaporean children (mean age: 13.96 ± 0.88 years) of different ethnicities (e.g., Chinese, Malay, and Indian). The corresponding value was –2.35 ± 2.49 D, with the cycloplegic refraction measured using an autokeratorefractometer [46]. These differences could be because we did not perform cycloplegic refraction measurements in our study, which might be a limitation. Finally, the differences might have resulted from differences in ethnicities and ages between studies.

The astigmatism vector analysis in the present study was significantly higher in children with ASD than in participants with TD. By contrast, Anketell et al. [25] reported that there was no significant difference in the magnitude of the cylindrical component between age groups for either children with ASD or typically developed children. Furthermore, Ezegwui et al. reported that 18 Nigerian children with ASD (mean age: 10.28 ± 3.20 years; range: 5–15 years) exhibited a significant incidence of astigmatism (22.2%) in the range of –1.00 to –2.00 DC; however, there were no comparisons with healthy children in their study [47]. Nevertheless, the results of both studies agreed with those of the present study in terms of the higher incidence of astigmatism in children with ASD.

The mean HVID of the participants with TD was comparable to that reported by Jiang et al. (12.02  ±  0.38 mm) for a large school-based population of children aged 4–18 years [48]. Matsuda et. al. reported that the mean corneal curvature of Asian and Caucasian eyes (age range: 16–60 years) was steeper than that reported in this study [49]. This may be due to differences in age range between studies, because with increasing age, the corneas become steeper. By contrast, an Asian study of corneal power compared typically developed children with a mean age of 12.4 ± 1.8 years and children with Down syndrome with a mean age of 12.8 ± 1.9 years. The corneal curvature was measured using a hand-held keratometer. The results revealed that the mean corneal curvature of typically developed children was flatter than the Down syndrome group [50]. This finding matched our results regarding the participants with TD. Overall, these variations in results may be due to differences in the characteristics of the specific populations, ages, and ethnicities.

The present study showed that there were no significant differences in corneal asphericity (Q-value) between children with ASD and participants with TD. The mean Q-values of the participants with TD (–0.11 ± 0.32) were higher than that obtained by Davis and co-workers (−0.346 ± 0.101) in their cross-sectional study examining a population with a mean age of 9.92 ± 2.42 years (range: 6–15 years). A lower Q-value indicates a less prolate corneal shape. In a prolate cornea, the radius of the periphery is larger than the radius of the curvature at the center. Davis et al. found that the myopic cornea was less prolate than hyperopic corneas [51]. 

There was an impact of patient’s collaboration on the measurements; three children were excluded from the study due to their poor cooperation for all the measurements including VA measurement, CS, refraction, and corneal topography. Only a 31 of the children with autism had completed the refraction and the corneal topography, except one patient with poor image quality of his right eye, who was excluded. Five children did not proceed to the VA test and seven did not pass CS due to their inability to concentrate. The poor collaboration of children in this study contributed to the result of a small sample size.

This study had some limitations, including the unbalanced gender recruitment of children with ASD, with 21 of 31 children (67.74%) being male. However, it is been previously reported that the prevalence of ASD and keratoconus are more frequent in males than in females [5,26]. On other hand, a more balanced gender ratio could provide in-depth insight of the risk of keratoconus in patients with ASD. In addition, the corneal diameter, which was used to calculate Q-values to represent corneal shape, was not measured using an Eye Sys Vista system. Furthermore, as refraction was measured without cycloplegia, hyperopia, and myopia, it might have been underestimated. Finally, the cooperation of some autism centers for the recruitment of children was not very good, resulting in a relatively small ASD sample size. In summary, this is the first study to report preliminary outcome of corneal topography in children with ASD and compare it with age-matched participants with TD. Most children with ASD had significant REs, and astigmatism was dominant in this group. Corneal astigmatism was significantly and inversely related to objective refractive astigmatism for both ASD and TD groups. Both corneal power (steep/flat-simulated K-readings and average simulated K-readings) and corneal shape descriptors (Q-value, IS, OSI, DSI, and CSI) did not differ significantly between children with ASD and participants with TD. This result could imply that individuals with ASD may not be more prone to develop keratoconus.

In conclusion, children with ASD are a special group that have interaction disabilities. Although there are challenges in examining this group of children, they nonetheless need to be provided with a high quality of eye care. They have a higher prevalence of REs, with astigmatism being the most common type [11,12,13,14,15]. The presence of undiagnosed or uncorrected REs is one of the causes of visual impairment [52,53]. Upon assessment of corneal topography in this preliminary report, no subclinical or clinical keratoconus was found in children with ASD at an early stage of life. This result could suggest that in the clinic, there is no crucial need to perform corneal topography measurements for each child with ASD. This would be time-consuming for children with ASD, parents, and optometrists, and would not be cost-efficient. 

Further investigations of larger samples of children with ASD and older participants are needed. Sample size calculation can be estimated on the basis of the prevalence of keratoconus in age groups, as suggested in previous studies [26,40,41]. The sample size could be calculated using specialized software such as the Epi-Info software (Centers for Disease Control and Prevention, Atlanta, GA, USA; http://www.cdc.gov/epiinfo/7/).

## 5. Conclusions

Both corneal power and corneal shape descriptors did not significantly differ between ASD and participants with TD. Thus, this preliminary result could indicate that it may not be crucial to conduct corneal topography measurements for teenage children with ASD.

## Figures and Tables

**Table 1:** The mean ± standard deviation of age, visual acuity, spherical equivalent, and cylindrical component in ASD participants and participants with TD. The statistical difference between both groups was investigated.

Variables	ASD Group Mean ± SD	TD Group Mean ± SD	Independent *t*-Test
Age (years)	12.78 ± 4.49 (*n* = 31)	13.65 ± 3.56 (*n* = 60)	t = −2.084, *p* = 0.075
Gender (male/female)	M = 21, F = 10	M = 33, F = 27	—
VA without correction (logMAR)	0.28 ± 0.29 (*n* = 26)	0.33 ± 0.37 (*n* = 60)	t = −0.35, *p* = 0.32
Spherical equivalent (diopters)	−0.76 ± 1.66 (*n* = 27)	0.96 ± 1.69 (*n* = 60)	t = 1.15, *p* = 0.15
Cylindrical component (diopters)	J_0_: −0.2 ± 0.47 J_45_: −0.35 ± 0.48 (*n* = 31)	J_0_: −0.10 ± 0.20 J_45_: −0.19 ± 0.18 (*n* = 60)	t = −1.22, *p* < 0.0001 * t = −0.54, *p* = 0.004 *

*p* < 0.05 indicates statistical significance. * Statistically significant. VA, visual acuity; TD, typical development participants.

**Table 2:** The refractive errors and contrast sensitivity of both eyes of children with autism spectrum disorder (ASD) and typical development (TD) participants.

Group Characteristics	ASD Group	TD Group
Emmtropia (*n*, %)	2 of 31, 6.45%	4 of 60, 6.6%
Simple myopia (*n*, %)	1 of 31, 3.22%	None
Anisometropia (*n*, %)	4 of 31, 12.90%	None
Compound hyperopic astigmatism (*n*, %)	9 of 31, 29.03%	17of 60, 28.33%
Compound myopic astigmatism (*n*, %)	8 of 31, 25.80%	27 of 60, 45%
Simple astigmatism (*n*, %)	5 of 31, 16.12%	10 of 60, 16.66%
Mixed astigmatism (*n*, %)	1 of 31, 3.22%	None
Spherical component (mean ± SD)	−0.13 ± 1.58	−0.67 ± 1.68
Cylindrical component (mean ± SD)	J_0_: −0.2 ± 0.47 J_45_: −0.35 ± 0.48	J_0_: −0.10 ± 0.2 J_45_: −0.19 ± 0.18
LogCS (mean ± SD)	OD: 1.61 ± 0.11 OU: 1.70 ± 0.08	OD: 1.65 ± 0.07 OU: 1.76 ± 0.05

SD, standard deviation; OD, right eye; OU, both eyes open; CS, contrast sensitivity.

**Table 3:** Summary of the mean ± standard deviation of corneal shape indices in ASD participants and participants with TD.

Variables	Children with ASD (Mean ± SD)	TD Participants (Mean ± SD)	Independent *t*-Test
PA (mm)	9.89 ± 1.04	10.78 ± 0.45	t = −6.859, *p* < 0.0001 *
HVID (mm)	11.44 ± 0.55	11.80 ± 0.40	t = −4.731, *p* = 0.003 *
Steep-K (diopters)	43.31 ± 2.39	43.10 ± 1.92	t = 075, *p* = 0.28
Flat-K (diopters)	42.21 ± 2.29	42.11 ± 1.96	t = 0.42, *p* = 0.33
Delta-K (diopters)	1.1 ± 0.59	0.99 ± 0.53	t = 1.33, *p* = 0.21
Average-K (diopters)	43.20 ± 2.36	42.59 ± 1.92	t = 1.12, *p* = 0.39
Q-value	−0.18 ± 0.36	−0.11 ± 0.32	t = −1.43, *p =* 0.38
IS	−0.24 ± 0.79	−0.17 ± 1.1	t = −0.88, *p* = 0.51
OSI	0.35 ± 0.54	0.20 ± 0.54	t = 1.12, *p* = 0.94
DSI	0.81 ± 0.81	0.63 ± 0.69	t = 0.96, *p* = 0.35
CSI	0.10 ± 0.03	0.11 ± 0.02	t = 0.17, *p* = 0.43

* p < 0.05 indicates statistical significance. ASD: autism spectrum disorder; TD: typical development participants; PA: vertical palpebral aperture; HVID: horizontal visible iris diameter; steep, flat, and average-K: simulated steep, flat, and average K-readings, respectively, for measuring corneal astigmatism; delta-K: corneal power; Q-value: corneal asphericity; IS: inferior–superior dioptric power; OSI: opposite sector index; DSI: differential sector index; CSI: center/surround index.

## Data Availability

The data presented in this study are available on request from the corresponding author.

## References

[1] American Psychiatric Association (2013). Diagnostic and Statistical Manual of Mental Disorders.

[2] Ozonoff S., Heung K., Byrd R., Hansen R., Hertz-Picciotto I. (2008). The onset of autism: Patterns of symptom emergence in the first years of life. Autism Res..

[3] Lord C., Risi S., DiLavore P.S., Shulman C., Thurm A., Pickles A. (2006). Autism from 2 to 9 years of age. Arch. Gen. Psychiatry.

[4] Anagnostou E., Zwaigenbaum L., Szatmari P., Fombonne E., Fernandez B.A., Woodbury-Smith M., Brian J., Bryson S., Smith I.M., Drmic I. (2014). Autism spectrum disorder: Advances in evidence-based practice. Cmaj.

[5] Zwaigenbaum L., Bryson S.E., Szatmari P., Brian J., Smith I.M., Roberts W., Vaillancourt T., Roncadin C. (2012). Sex Differences in Children with Autism Spectrum Disorder Identified Within a High-Risk Infant Cohort. J. Autism Dev. Disord..

[6] Al-Salehi S.M., Al-Hifthy E.H., Ghaziuddin M. (2009). Autism in Saudi Arabia: Presentation, Clinical Correlates and Comorbidity. Transcult. Psychiatry.

[7] Hedley D., Young R., Brewer N. (2012). Using Eye Movements as an Index of Implicit Face Recognition in Autism Spectrum Disorder. Autism Res..

[8] Wegiel J., Kuchna I., Nowicki K., Imaki H., Wegiel J., Ma S.Y., Azmitia E.C., Banerjee P., Flory M., Cohen I.L. (2013). Contribution of olivofloccular circuitry developmental defects to atypical gaze in autism. Brain Res..

[9] Rosenhall U., Johansson E., Gillberg C. (1988). Oculomotor findings in autistic children. J. Laryngol. Otol..

[10] Wilkes B.J., Carson T.B., Patel K.P., Lewis M.H., White K.D. (2015). Oculomotor performance in children with high-functioning Autism Spectrum Disorders. Res. Dev. Disabil..

[11] Leat S.J., Mohr A. (2007). Accommodative Response in Pre-presbyopes with Visual Impairment and Its Clinical Implications. Investig. Ophthalmol. Vis. Sci..

[12] Millodot M. (2015). The effect of refractive error on the accommodative response gradient: A summary and update. Ophthalmic Physiol. Opt..

[13] Dickinson C. (1998). Low Vision: Principles and Practice.

[14] Schwiegerling J., Greivenkamp J.E. (1996). Keratoconus detection based on videokeratoscopic height data. Optom. Vis. Sci..

[15] Albrecht M.A., Stuart G.W., Falkmer M., Ordqvist A., Leung D., Foster J.K., Falkmer T. (2014). Brief Report: Visual Acuity in Children with Autism Spectrum Disorders. J. Autism Dev. Disord..

[16] Anketell P.M., Saunders K.J., Gallagher S.M., Bailey C., Little J.-A. (2015). Brief Report: Vision in Children with Autism Spectrum Disorder: What Should Clinicians Expect?. J. Autism Dev. Disord..

[17] Manh V., Chen A.M., Tarczy-Hornoch K., Cotter S.A., Candy T.R. (2015). Accommodative Performance of Children with Unilateral Amblyopia. Investig. Ophtalmol. Vis. Sci..

[18] Behrmann M., Thomas C., Humphreys K. (2006). Seeing it differently: Visual processing in autism. Trends Cogn. Sci..

[19] Bertone A., Mottron L., Jelenic P., Faubert J. (2005). Enhanced and diminished visuo-spatial information processing in autism depends on stimulus complexity. Brain.

[20] Davis R.A.O., Bockbrader M.A., Murphy R.R., Hetrick W.P., O’Donnell B.F. (2006). Subjective Perceptual Distortions and Visual Dysfunction in Children with Autism. J. Autism Dev. Disord..

[21] Guy J., Mottron L., Berthiaume C., Bertone A. (2016). The developmental trajectory of contrast sensitivity in autism spectrum disorder. Autism Res..

[22] Franklin A., Sowden P., Burley R., Notman L., Alder E. (2008). Color perception in children with autism. J. Autism Dev. Disord..

[23] Rhodes G., Ewing L., Jeffery L., Avard E., Taylor L. (2014). Reduced adaptability, but no fundamental disruption, of norm-based face-coding mechanisms in cognitively able children and adolescents with autism. Neuropsychologia.

[24] Denis D., Burillon C., Livet M.O., Burguiere O. (1997). Ophthalmologic signs in children with autism. J. Fr. Ophtalmol..

[25] Anketell P.M., Saunders K.J., Gallagher S., Bailey C., Little J.A. (2016). Profile of refractive errors in European Caucasian children with Autistic Spectrum Disorder; increased prevalence and magnitude of astigmatism. Ophthalmic Physiol. Opt..

[26] Hashemi H., Heydarian S., Hooshmand E., Saatchi M., Yekta A., Aghamirsalim M., Valadkhan M., Mortazavi M., Hashemi A., Khabazkhoob M. (2020). The Prevalence and Risk Factors for Keratoconus: A Systematic Review and Meta-Analysis. Cornea.

[27] Lindsay R., Smith G., Atchison D.A. (1998). Descriptors of Corneal Shape. Optom. Vis. Sci..

[28] Cavas-Martínez F., De la Cruz Sánchez E., Nieto Martínez J., Fernández Cañavate F.J., Fernández-Pacheco D.G. (2016). Corneal topography in keratoconus: State of the art. Eye Vis..

[29] Maeda N., Klyce S.D., Smolek M.K., Thompson H.W. (1994). Automated keratoconus screening with corneal topography analysis. Investig. Ophthalmol. Vis. Sci..

[30] Carlson N., Kurtz D. (2015). Clinical Procedures for Ocular Examination.

[31] Dougherty B.E., Flom R.E., Bullimore M.A. (2005). An Evaluation of the Mars Letter Contrast Sensitivity Test. Optom. Vis. Sci..

[32] Yalcin E., Sultan P., Yilmaz S., Pallikaris I.G. (2017). A Comparison of Refraction Defects in Childhood Measured Using Plusoptix S09, 2WIN Photorefractometer, Benchtop Autorefractometer, and Cycloplegic Retinoscopy. Semin. Ophthalmol..

[33] Thibos L.N., Wheeler W., Horner D. (1997). Power Vectors: An Application of Fourier Analysis to the Description and Statistical Analysis of Refractive Error. Optom. Vis. Sci..

[34] EyeSys Vista. http://eyesys.com/index.html.

[35] Wang Q., Savini G., Hoffer K.J., Xu Z., Feng Y., Wen D., Hua Y., Yang F., Pan C., Huang J. (2012). A Comprehensive Assessment of the Precision and Agreement of Anterior Corneal Power Measurements Obtained Using 8 Different Devices. PLoS ONE.

[36] Bergmanson J.P., Martinez J.G. (2017). Size does matter: What is the corneo-limbal diameter?. Clin. Exp. Optom..

[37] VasanthaKumar P., Kumar P., Rao M. (2013). Anthropometric Analysis of Palpebral Fissure Dimensions and its Position in South Indian Ethnic Adults. Oman Med. J..

[38] Montalbán R., Piñero D.P., Javaloy J., Alió J.L. (2013). Correlation of the corneal toricity between anterior and posterior corneal surfaces in the normal human eye. Cornea.

[39] Montalbán R., Piñero D.P., Javaloy J., Alió J.L. (2012). Scheimpflug photography–based clinical characterization of the correlation of the corneal shape between the anterior and posterior corneal surfaces in the normal human eye. J. Cataract Refract. Surg..

[40] Godefrooij D.A., De Wit G.A., Uiterwaal C.S., Imhof S.M., Wisse R.P.L. (2017). Age-specific Incidence and Prevalence of Keratoconus: A Nationwide Registration Study. Am. J. Ophthalmol..

[41] Netto E.A.T., Al-Otaibi W.M., Hafezi N.L., Kling S., Al-Farhan H.M., Randleman J.B., Hafezi F. (2018). Prevalence of keratoconus in paediatric patients in Riyadh, Saudi Arabia. Br. J. Ophthalmol..

[42] Helsmoortel C., Silfhout A.T.V.-V., Coe B.P., Vandeweyer G., Rooms L., Ende J.V.D., Schuurs-Hoeijmakers J.H., Marcelis C.L., Willemsen M.H., Vissers L.E. (2014). A SWI/SNF-related autism syndrome caused by de novo mutations in ADNP. Nat. Genet..

[43] Read S.A., Collins M.J., Carney L.G., Iskander D.R. (2006). The morphology of the palpebral fissure in different directions of vertical gaze. Optom. Vis. Sci..

[44] Léoni-Mesplié S., Mortemousque B., Mesplié N., Touboul D., Praud D., Malet F., Colin J. (2012). Epidemiological aspects of keratoconus in children. J. Fr. Ophtalmol..

[45] Chen M.-H., Su T.-P., Chen Y.-S., Hsu J.-W., Huang K.-L., Chang W.-H., Chen T.-J., Pan T.-L., Bai Y.-M. (2014). Is atopy in early childhood a risk factor for ADHD and ASD? A longitudinal study. J. Psychosom. Res..

[46] Lim L., Gazzard G., Chan Y.-H., Fong A., Kotecha A., Sim E.-L., Tan D., Tong L., Saw S.-M. (2008). Cornea Biomechanical Characteristics and Their Correlates with Refractive Error in Singaporean Children. Investig. Ophthalmol. Vis. Sci..

[47] Ezegwui I., Lawrence L., Aghaji A., Okoye O., Onwasigwe E., Ebigbo P. (2014). Refractive errors in children with autism in a developing country. Niger. J. Clin. Pr..

[48] Jiang W.J., Wu H., Wu J.F., Hu Y.Y., Lu T.L., Sun W., Guo D.D., Wang X.R., Bi H., Jonas J.B. (2016). Corneal diameter and associated parameters in Chinese children: The Shandong Children Eye Study. Clin. Exp. Ophthalmol..

[49] Matsuda L.M., Woldorff C.L., Kame R.T., Hayashida J.K. (1992). Clinical comparison of corneal diameter and curvature in Asian eyes with those of Caucasian eyes. Optom. Vis. Sci..

[50] Little J., Woodhouse J.M., Saunders K.J. (2009). Corneal Power and Astigmatism in Down Syndrome. Optom. Vis. Sci..

[51] Davis W.R., Raasch T.W., Mitchell G.L., Mutti D.O., Zadnik K. (2005). Corneal asphericity and apical curvature in children: A cross-sectional and longitudinal evaluation. Investig. Ophthalmol. Vis. Sci..

[52] Pascolini D., Mariotti S.P. (2012). Global estimates of visual impairment: 2010. Br. J. Ophthalmol..

[53] Congdon N.G., O’Colmain B., Klaver C.C.W., Klein R., Muñoz B., Friedman D.S., Kempen J., Taylor H.R., Mitchell P., Eye Diseases Prevalence Research Group (2004). Causes and Prevalence of Visual Impairment Among Adults in the United States. Arch. Ophthalmol..

